# Supplementary material for: Influenza A Viruses in Ruddy Turnstones (Arenaria interpres); Connecting Wintering and Migratory Sites with an Ecological Hotspot at Delaware Bay
Source: Viruses. 2020 Oct 22;12(11):1205. doi: 10.3390/v12111205 (PMC7690596; doi:10.3390/v12111205)
Supplement: Supplementary file 1 [file viruses-12-01205-s001.pdf]

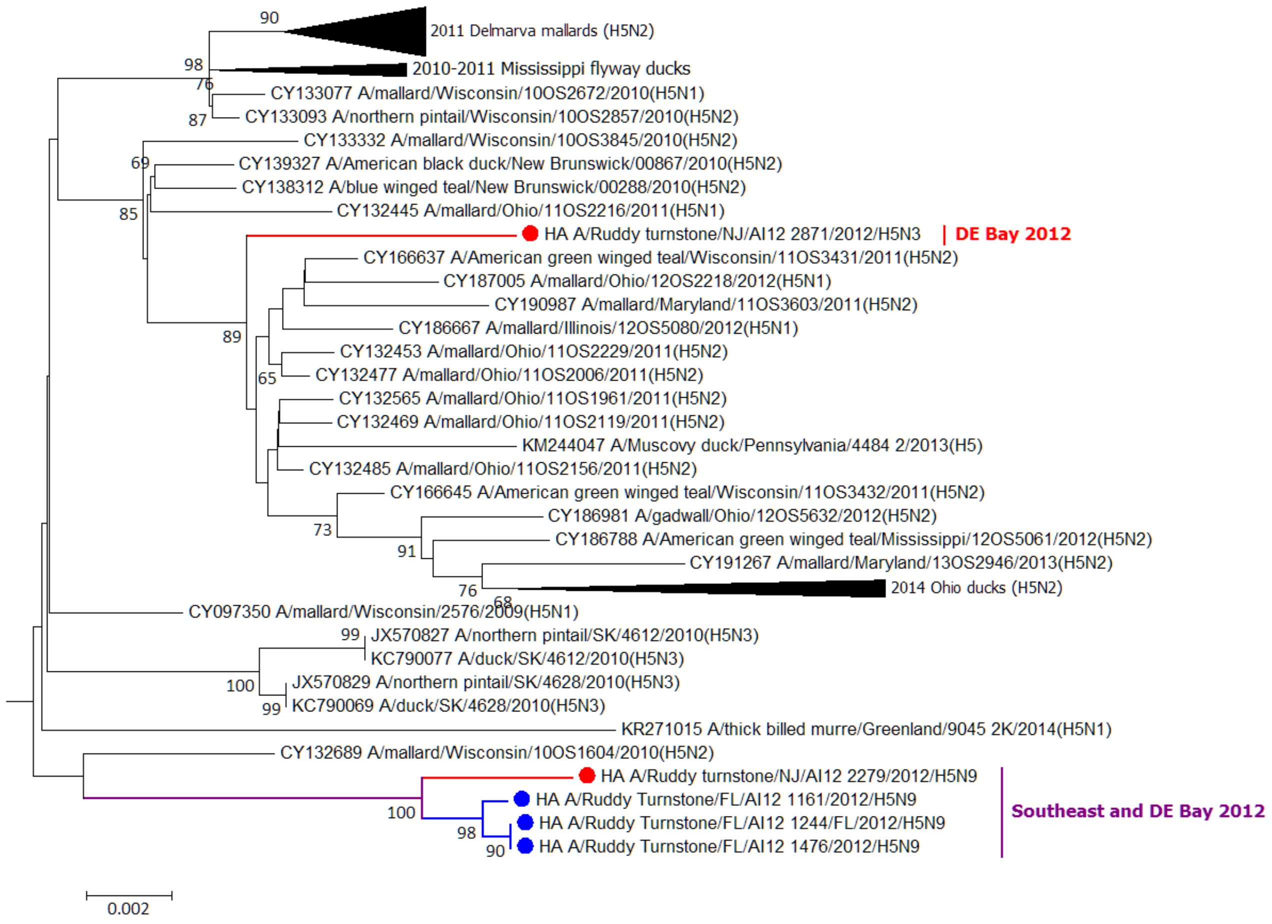

Supplemental Figure S1. Maximum-likelihood (ML) phylogenetic sub-tree for hemagglutinin HA5 gene segments derived from influenza A viruses (IAV) isolated from wild and domestic birds in North and South America (excluding Alaska), without date restriction. Nodes for HA5 segments identified in this study are colored in red (Delaware Bay) or blue (southeast) circles. Bootstrap values lower than 65 are omitted. Branch lengths are measured in the number of nucleotide substitutions per site.

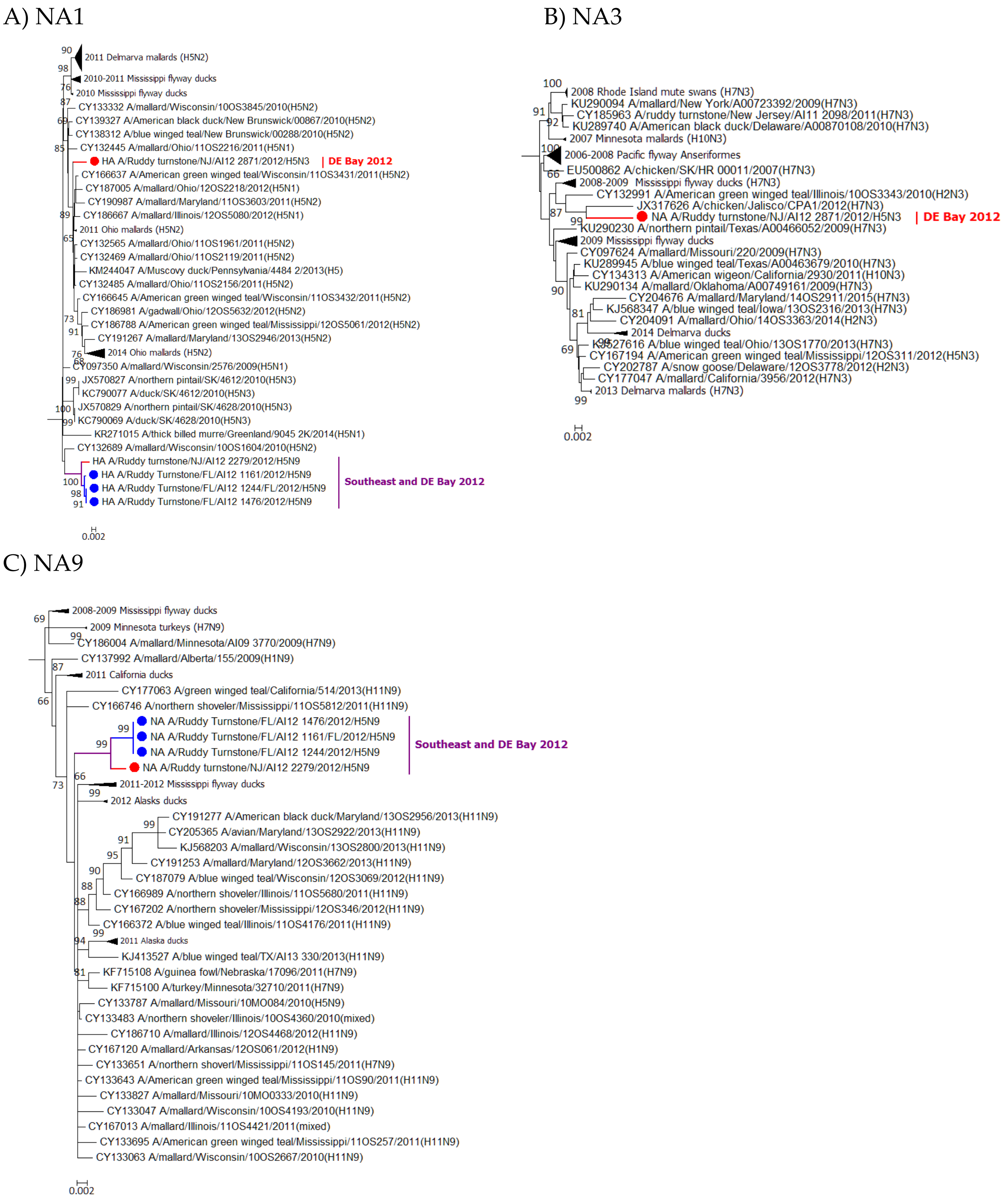

Supplemental Figure S2. Maximum-likelihood (ML) phylogenetic sub-trees for neuraminidase (NA) gene segments derived from IAV isolated from wild and domestic birds in N. and S. America without date restriction. Nodes for NA segments identified in this study are colored in red (DE Bay) or blue (southeast) markers. Branch lengths are measured in the number of nucleotide substitutions per site Bootstrap values lower than 65 are omitted. (A) NA1; (B) NA3; (C) NA9.

A)

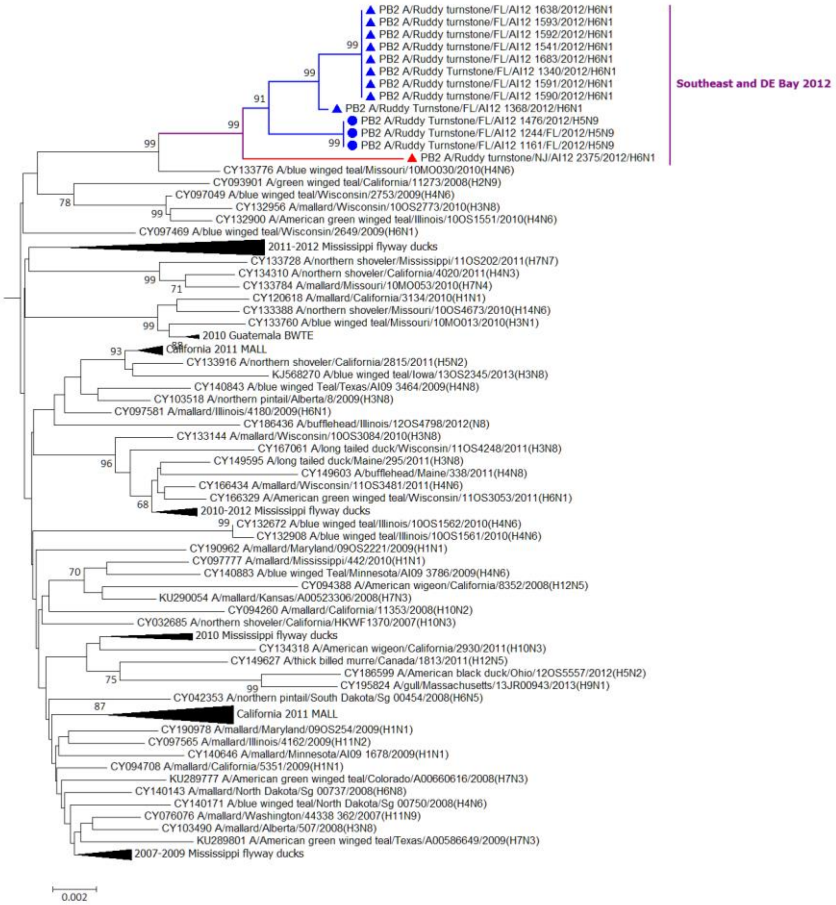

B)

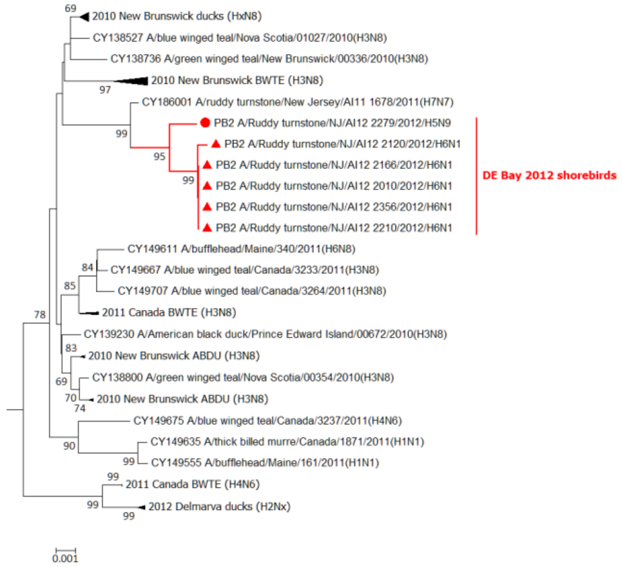

Supplemental Figure S3. Maximum-likelihood (ML) phylogenetic sub-trees for PB2 gene segments derived from IAV isolated from wild and domestic birds in N. and S. America with date restriction (2000 – 2015). Nodes for PB2 segments identified in this study are colored in red (DE Bay) or blue (southeast) markers. Branch lengths are measured in the number of nucleotide substitutions per site Bootstrap values lower than 65 are omitted. A) Southeast and one DE Bay sequence; B) Clade of DE Bay PB2 sequences.

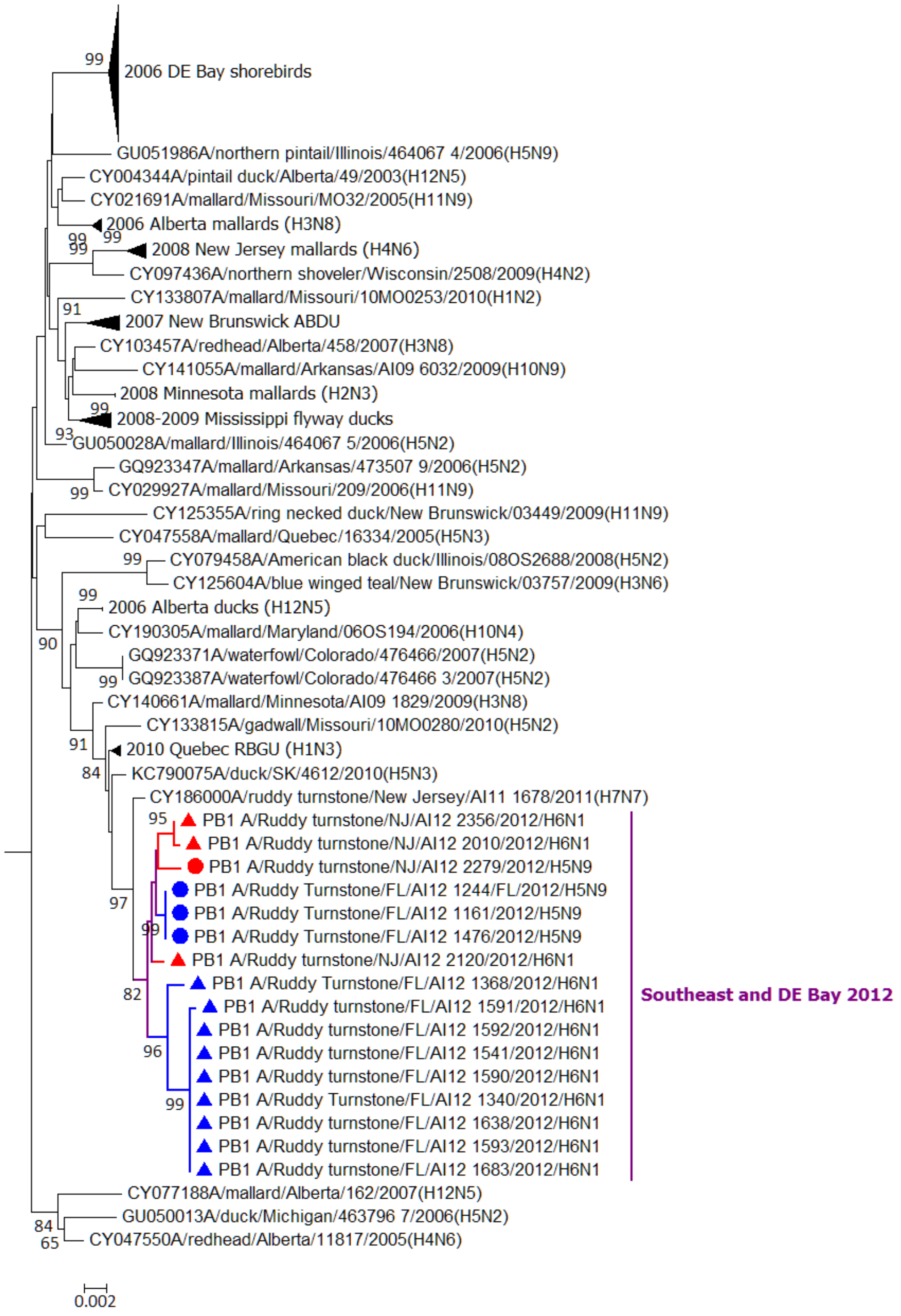

Supplemental Figure S4. Maximum-likelihood (ML) phylogenetic sub-tree for PB1 gene segments derived from IAV isolated from wild and domestic birds in N. and S. America with date restriction (2000 – 2015). Nodes for most PB2 segments identified in this study are colored in red (DE Bay) or blue (southeast) markers. Branch lengths are measured in the number of nucleotide substitutions per site Bootstrap values lower than 65 are omitted.

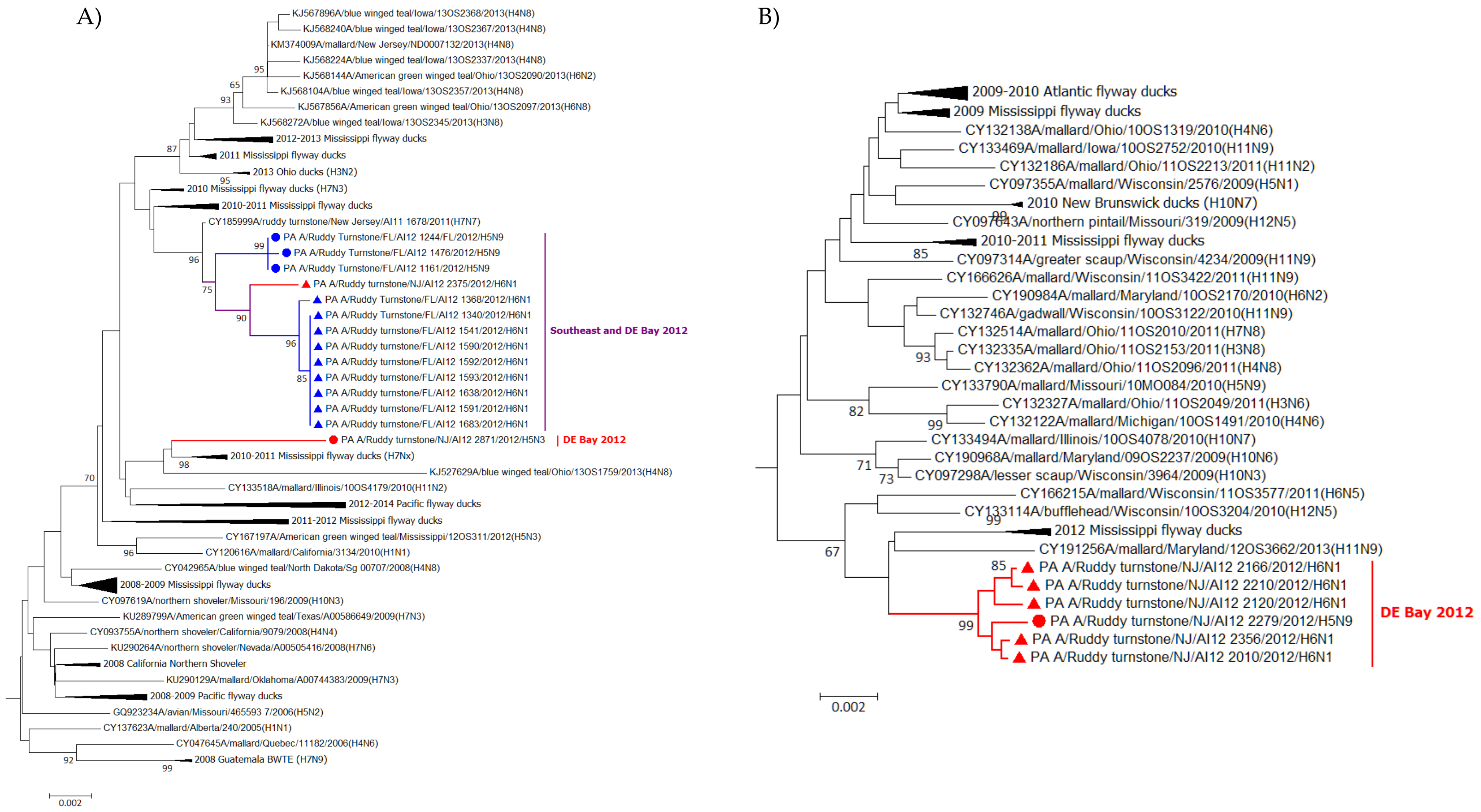

Supplemental Figure S5. Maximum-likelihood (ML) phylogenetic sub-tree for PA gene segments derived from IAV isolated from wild and domestic birds in N. and S. America with date restriction (2000 – 2015). Nodes for PA segments identified in this study are colored in red (DE Bay) or blue (southeast) markers. Branch lengths are measured in the number of nucleotide substitutions per site. Bootstrap values lower than 65 are omitted. A) Southeast and DE Bay PA gene sequences; B) Clade of DE Bay PA gene sequences.

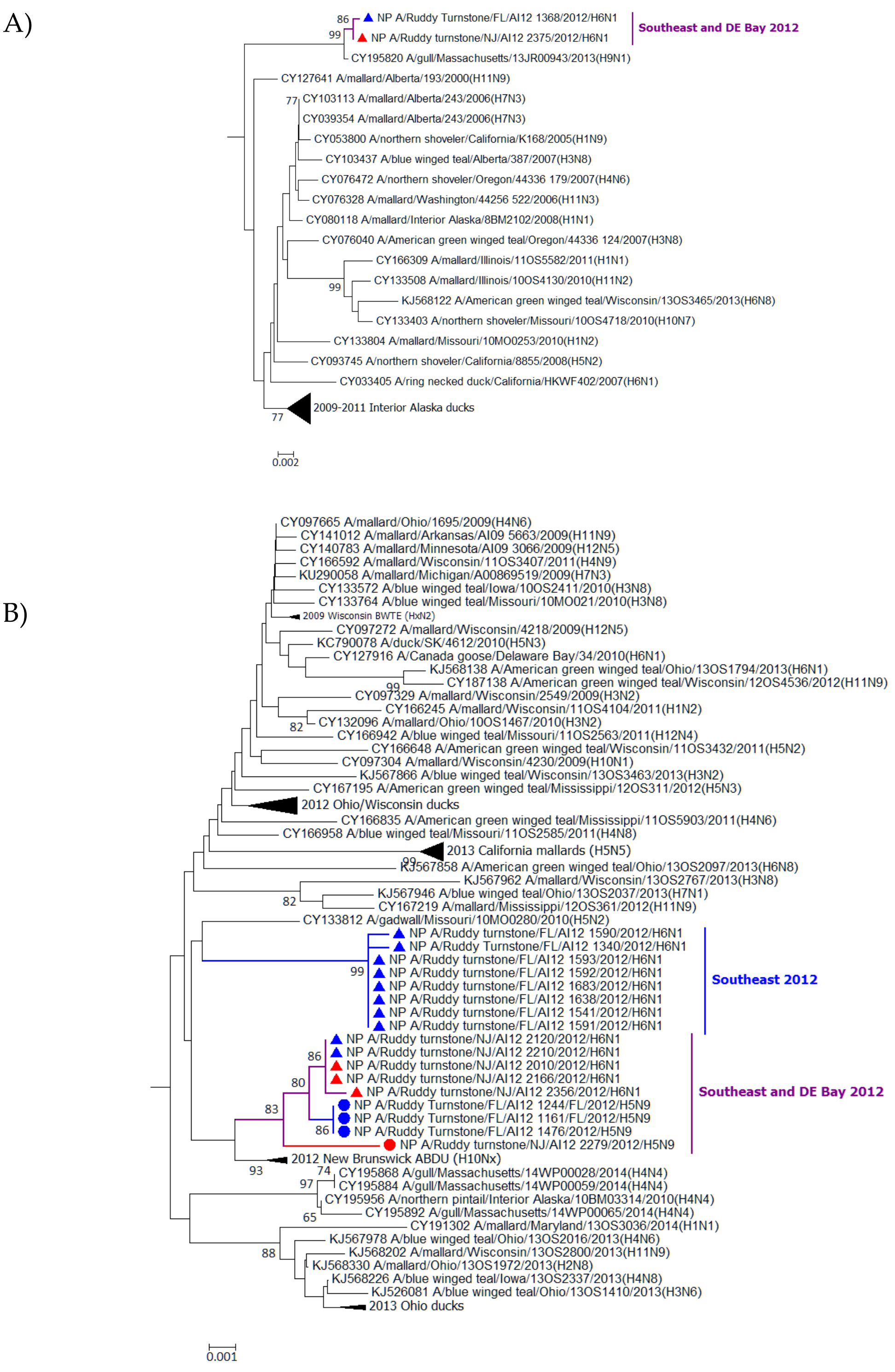

Supplemental Figure S6. Maximum-likelihood (ML) phylogenetic sub-trees for nucleoprotein (NP) gene segments derived from IAV isolated from wild and domestic birds in N. and S. America with date restriction (2000 – 2015). Nodes for NP segments identified in this study are colored in red (DE Bay) or blue (southeast) markers. Branch lengths are measured in the number of nucleotide substitutions per site. Bootstrap values lower than 65 are omitted.

A) Clade of one southeast and one and DE Bay NP sequence; B) Southeast and DE Bay NP sequences.

A)

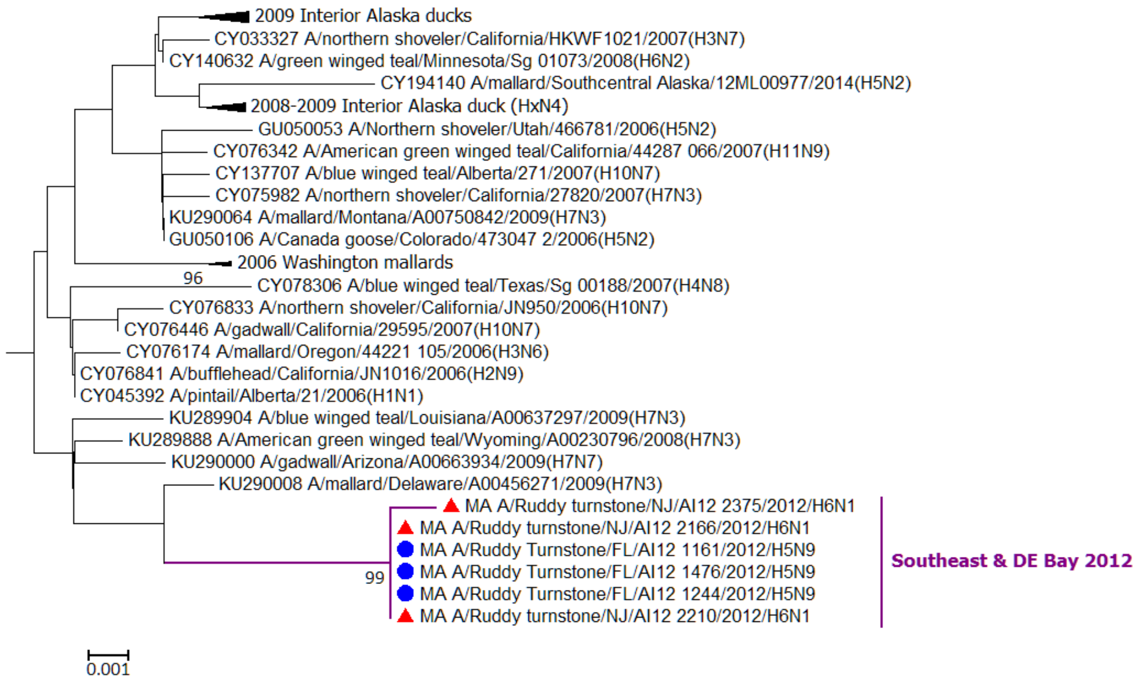

2009 Interior Alaska ducks
CY033327 A/northern shoveler/California/HKWF1021/2007(H3N7)
CY140632 A/green winged teal/Minnesota/Sg 01073/2008(H6N2)
CY194140 A/mallard/Southcentral Alaska/12ML00977/2014(H5N2)
2008-2009 Interior Alaska duck (HxN4)
GU050053 A/Northern shoveler/Utah/466781/2006(H5N2)
CY076342 A/American green winged teal/California/44287 066/2007(H11N9)
CY137707 A/blue winged teal/Alberta/271/2007(H10N7)
CY075982 A/northern shoveler/California/27820/2007(H7N3)
KU290064 A/mallard/Montana/A00750842/2009(H7N3)
GU050106 A/Canada goose/Colorado/473047 2/2006(H5N2)
2006 Washington mallards
96
CY078306 A/blue winged teal/Texas/Sg 00188/2007(H4N8)
CY076833 A/northern shoveler/California/JN950/2006(H10N7)
CY076446 A/gadwall/California/29595/2007(H10N7)
CY076174 A/mallard/Oregon/44221 105/2006(H3N6)
CY076841 A/bufflehead/California/JN1016/2006(H2N9)
CY045392 A/pintail/Alberta/21/2006(H1N1)
KU289904 A/blue winged teal/Louisiana/A00637297/2009(H7N3)
KU289888 A/American green winged teal/Wyoming/A00230796/2008(H7N3)
KU290000 A/gadwall/Arizona/A00663934/2009(H7N7)
KU290008 A/mallard/Delaware/A00456271/2009(H7N3)
MA A/Ruddy turnstone/NJ/AI12 2375/2012/H6N1
MA A/Ruddy turnstone/NJ/AI12 2166/2012/H6N1
MA A/Ruddy Turnstone/FL/AI12 1161/2012/H5N9
MA A/Ruddy Turnstone/FL/AI12 1476/2012/H5N9
MA A/Ruddy Turnstone/FL/AI12 1244/2012/H5N9
MA A/Ruddy turnstone/NJ/AI12 2210/2012/H6N1
99
Southeast & DE Bay 2012
0.001

B)

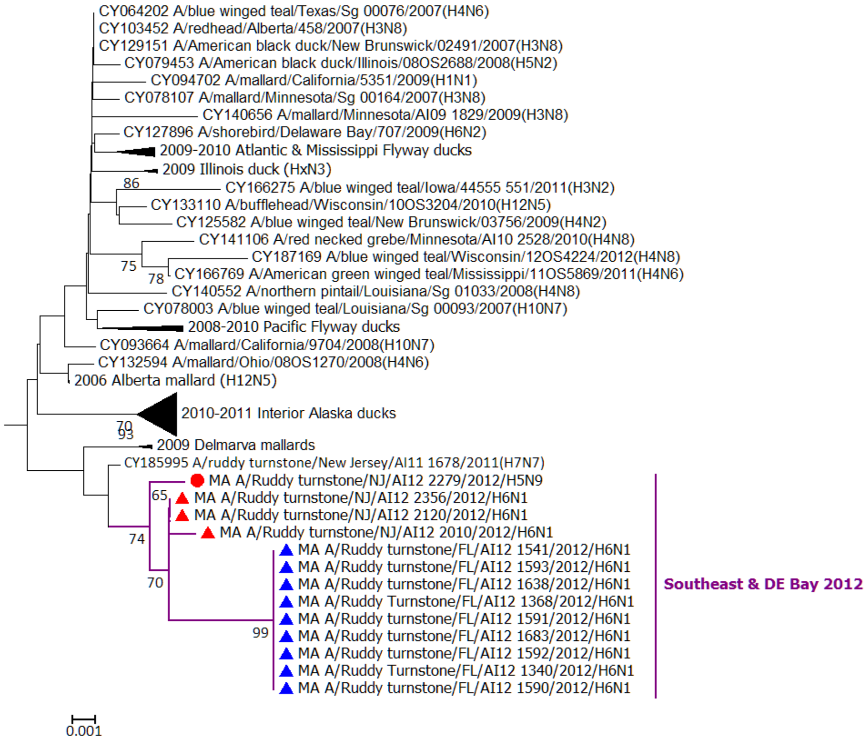

Supplemental Figure S7. Maximum-likelihood (ML) phylogenetic sub-trees for matrix (MA) gene segments derived from IAV isolated from wild and domestic birds in N. and S. America with date restriction (2000 – 2015). Nodes for MA segments identified in this study are colored in red (DE Bay) or blue (southeast) markers. Branch lengths are measured in the number of nucleotide substitutions per site. Bootstrap values lower than 65 are omitted. A) and B) Matrix genes identified here fall out into one of two distinct clades.

A)

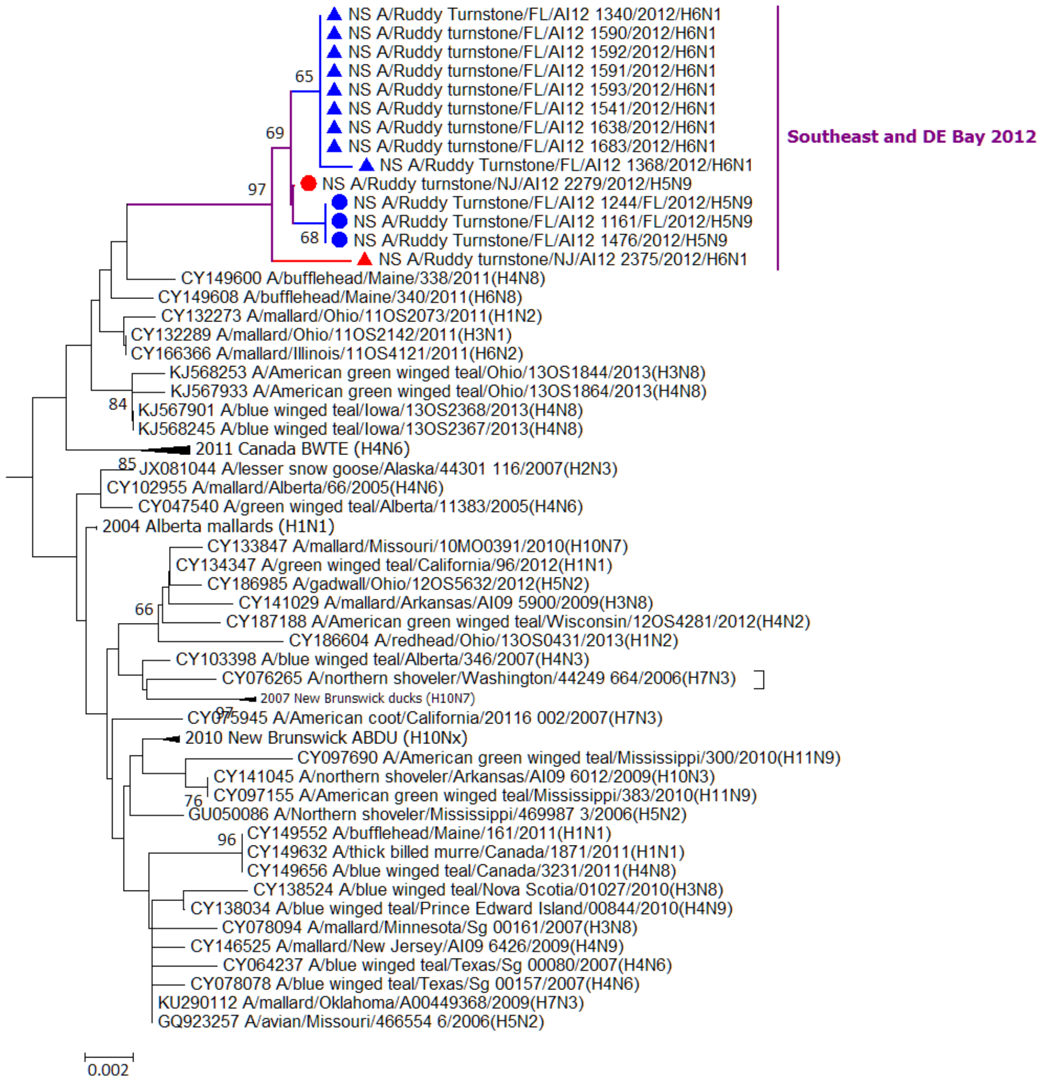
NS A/Ruddy Turnstone/FL/AI12 1340/2012/H6N1
NS A/Ruddy turnstone/FL/AI12 1590/2012/H6N1
NS A/Ruddy turnstone/FL/AI12 1592/2012/H6N1
NS A/Ruddy turnstone/FL/AI12 1591/2012/H6N1
NS A/Ruddy turnstone/FL/AI12 1593/2012/H6N1
NS A/Ruddy turnstone/FL/AI12 1541/2012/H6N1
NS A/Ruddy turnstone/FL/AI12 1638/2012/H6N1
NS A/Ruddy turnstone/FL/AI12 1683/2012/H6N1
NS A/Ruddy Turnstone/FL/AI12 1368/2012/H6N1
NS A/Ruddy turnstone/NJ/AI12 2279/2012/H5N9
NS A/Ruddy Turnstone/FL/AI12 1244/FL/2012/H5N9
NS A/Ruddy Turnstone/FL/AI12 1161/FL/2012/H5N9
NS A/Ruddy Turnstone/FL/AI12 1476/2012/H5N9
NS A/Ruddy turnstone/NJ/AI12 2375/2012/H6N1
Southeast and DE Bay 2012
65
69
97
68
CY149600 A/bufflehead/Maine/338/2011(H4N8)
CY149608 A/bufflehead/Maine/340/2011(H6N8)
CY132273 A/mallard/Ohio/11OS2073/2011(H1N2)
CY132289 A/mallard/Ohio/11OS2142/2011(H3N1)
CY166366 A/mallard/Illinois/11OS4121/2011(H6N2)
KJ568253 A/American green winged teal/Ohio/13OS1844/2013(H3N8)
KJ567933 A/American green winged teal/Ohio/13OS1864/2013(H4N8)
84
KJ567901 A/blue winged teal/Iowa/13OS2368/2013(H4N8)
KJ568245 A/blue winged teal/Iowa/13OS2367/2013(H4N8)
2011 Canada BWTE (H4N6)
85
JX081044 A/lesser snow goose/Alaska/44301 116/2007(H2N3)
CY102955 A/mallard/Alberta/66/2005(H4N6)
CY047540 A/green winged teal/Alberta/11383/2005(H4N6)
2004 Alberta mallards (H1N1)
CY133847 A/mallard/Missouri/10MO0391/2010(H10N7)
CY134347 A/green winged teal/California/96/2012(H1N1)
CY186985 A/gadwall/Ohio/12OS5632/2012(H5N2)
CY141029 A/mallard/Arkansas/AI09 5900/2009(H3N8)
66
CY187188 A/American green winged teal/Wisconsin/12OS4281/2012(H4N2)
CY186604 A/redhead/Ohio/13OS0431/2013(H1N2)
CY103398 A/blue winged teal/Alberta/346/2007(H4N3)
CY076265 A/northern shoveler/Washington/44249 664/2006(H7N3)
2007 New Brunswick ducks (H10N7)
97
A/American coot/California/20116 002/2007(H7N3)
2010 New Brunswick ABDU (H10Nx)
CY097690 A/American green winged teal/Mississippi/300/2010(H11N9)
CY141045 A/northern shoveler/Arkansas/AI09 6012/2009(H10N3)
76
CY097155 A/American green winged teal/Mississippi/383/2010(H11N9)
GU050086 A/Northern shoveler/Mississippi/469987 3/2006(H5N2)
96
CY149552 A/bufflehead/Maine/161/2011(H1N1)
CY149632 A/thick billed murre/Canada/1871/2011(H1N1)
CY149656 A/blue winged teal/Canada/3231/2011(H4N8)
CY138524 A/blue winged teal/Nova Scotia/01027/2010(H3N8)
CY138034 A/blue winged teal/Prince Edward Island/00844/2010(H4N9)
CY078094 A/mallard/Minnesota/Sg 00161/2007(H3N8)
CY146525 A/mallard/New Jersey/AI09 6426/2009(H4N9)
CY064237 A/blue winged teal/Texas/Sg 00080/2007(H4N6)
CY078078 A/blue winged teal/Texas/Sg 00157/2007(H4N6)
KU290112 A/mallard/Oklahoma/A00449368/2009(H7N3)
GQ923257 A/avian/Missouri/466554 6/2006(H5N2)
0.002

B)

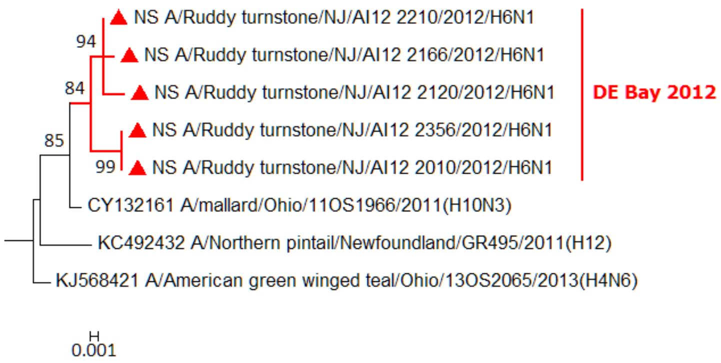

Supplemental Figure S8. Maximum-likelihood (ML) phylogenetic sub-trees for non-structural (NS) gene segments derived from IAV isolated from wild and domestic birds in N. and S. America with date restriction (2000 – 2015). Nodes for NP segments identified in this study are colored in red (DE Bay) or blue (southeast) markers. Branch lengths are measured in the number of nucleotide substitutions per site. Bootstrap values lower than 65 are omitted. A) Southeast and DE Bay NS gene sequences; B) Clade of DE Bay NS gene sequences.

Supplemental Table S1. GenBank accession numbers for viruses isolated and sequenced as part of this study.

Virus	GenBank Accession Number							
	PB2	PB1	PA	HA	NP	NA	MA	NS
A/Ruddy turnstone/Florida/AI12-1161/2012/H5N9	MW055318	MW055314	MW055288	MW055287	MW055316	MW055315	MW055313	MW055317
A/Ruddy turnstone/Florida/AI12-1244/2012/H5N9	MW055279	MW055275	MW055353	MW055352	MW055277	MW055276	MW055274	MW055278
A/Ruddy turnstone/Florida/AI12-1476/2012/H5N9	MW055357	MW055359	MW055394	MW055395	MW055356	MW055354	MW055355	MW055358
A/Ruddy turnstone/Florida/AI12-1590/2012/H6N1	MW055338	MW055341	MW055393	MW055342	MW055340	MW055337	MW055339	MW055336
A/Ruddy turnstone/Florida/AI12-1591/2012/H6N1	MW055404	MW055406	MW055366	MW055407	MW055408	MW055405	MW055409	MW055403
A/Ruddy turnstone/Florida/AI12-1592/2012/H6N1	MW055300	MW055303	MW055328	MW055298	MW055302	MW055304	MW055299	MW055301
A/Ruddy turnstone/Florida/AI12-1593/2012/H6N1	MW055293	MW055295	MW055312	MW055294	MW055291	MW055292	MW055296	MW055297
A/Ruddy turnstone/Florida/AI12-1683/2012/H6N1	MW055376	MW055377	MW055392	MW055374	MW055373	MW055375	MW055372	MW055371
A/Ruddy turnstone/Florida/AI12-1638/2012/H6N1	MW055347	MW055343	MW055424	MW055345	MW055348	MW055349	MW055344	MW055346
A/Ruddy turnstone/Florida/AI12-1340/2012/H6N1	MW055283	MW055286	MW055265	MW055284	MW055281	MW055282	MW055285	MW055280
A/Ruddy turnstone/Florida/AI12-1368/2012/H6N1	MW055334	MW055331	MW055378	MW055333	MW055330	MW055329	MW055332	MW055335
A/Ruddy turnstone/New Jersey/AI12-2120/2012/H6N1	MW055417	MW055423	MW055264	MW055418	MW055419	MW055422	MW055421	MW055420
A/Ruddy turnstone/New Jersey/AI12-2279/2012/H5N9	MW055360	MW055364	MW055350	MW055351	MW055363	MW055361	MW055365	MW055362
A/Ruddy turnstone/New Jersey/AI12-2166/2012/H6N1	MW055267	MW055270	MW055290	MW055272	MW055269	MW055271	MW055266	MW055268
A/Ruddy turnstone/New Jersey/AI12-2356/2012/H6N1	MW055389	MW055391	MW055273	MW055388	MW055385	MW055386	MW055390	MW055387
A/Ruddy turnstone/New Jersey/AI12-2375/2012/H6N1	MW055401	MW055399	MW055289	MW055400	MW055396	MW055402	MW055397	MW055398
A/Ruddy turnstone/New Jersey/AI12-2871/2012/H5N3	MW055380	MW055379	MW055326	MW055327	MW055384	MW055383	MW055381	MW055382
A/Ruddy turnstone/New Jersey/AI12-2010/2012/H6N1	MW055321	MW055323	MW055370	MW055319	MW055322	MW055325	MW055320	MW055324

A/Ruddy turnstone/New Jersey/AI12-2210/2012/H6N1	MW055412	MW055411	MW055369	MW055413	MW055416	MW055414	MW055415	MW055410

Supplemental Table S2. Pairwise distance matrix for 2,275 nucleotides of the PB2 gene (segment 1) for viruses analyzed in this study, and two reference sequences. Values are in percentages and shaded according to nucleotide identity: dark gray ≥ 99.0%; light gray ≥ 97.0%.

			**Southeastern US viruses**												**Delaware Bay viruses**								**Ref**
**Subtype**	**Season**	**Virus ID**	**1161[d]**	**1244**	**1340**	**1368**	**1476**	**1541**	**1590**	**1591**	**1592**	**1593**	**1638**	**1683**	**2010**	**2120**	**2166**	**2210**	**2279**	**2356**	**2375**	**2871**	**CY186001**
H5N9	ES[a]	1244	100.0																				
H6N1	LS[b]	1340	99.3	99.3																			
H6N1	LS	1368	99.4	99.4	99.8																		
H5N9	ES	1476	100.0	100.0	99.3	99.4																	
H6N1	LS	1541	99.3	99.3	100.0	99.8	99.3																
H6N1	LS	1590	99.3	99.3	100.0	99.7	99.3	100.0															
H6N1	LS	1591	99.3	99.3	100.0	99.8	99.3	100.0	100.0														
H6N1	LS	1592	99.3	99.3	100.0	99.8	99.3	100.0	100.0	100.0													
H6N1	LS	1593	99.3	99.3	100.0	99.8	99.3	100.0	100.0	100.0	100.0												
H6N1	LS	1638	99.3	99.3	100.0	99.8	99.3	100.0	100.0	100.0	100.0	100.0											
H6N1	LS	1683	99.3	99.3	100.0	99.8	99.3	100.0	100.0	100.0	100.0	100.0	100.0										
H6N1	DB[c]	2010	91.8	91.8	91.7	91.8	91.8	91.7	91.7	91.7	91.7	91.7	91.7	91.7									
H6N1	DB	2120	91.8	91.8	91.7	91.9	91.8	91.7	91.8	91.7	91.7	91.7	91.7	91.7	100.0								
H6N1	DB	2166	91.8	91.8	91.7	91.8	91.8	91.7	91.7	91.7	91.7	91.7	91.7	91.7	100.0	100.0							
H6N1	DB	2210	91.8	91.8	91.7	91.8	91.8	91.7	91.7	91.7	91.7	91.7	91.7	91.7	100.0	100.0	100.0						
H5N9	DB	2279	91.8	91.8	91.7	91.8	91.8	91.7	91.7	91.7	91.7	91.7	91.7	91.7	99.7	99.7	99.7	99.7					
H6N1	DB	2356	91.8	91.8	91.7	91.9	91.8	91.7	91.8	91.7	91.7	91.7	91.7	91.7	100.0	99.9	100.0	100.0	99.7				
H6N1	DB	2375	99.0	99.0	99.0	99.0	99.0	99.0	99.0	99.0	99.0	99.0	99.0	99.0	91.6	91.6	91.6	91.6	91.6	91.6			
H5N3	DB	2871	91.5	91.5	91.4	91.6	91.5	91.4	91.5	91.4	91.4	91.4	91.4	91.4	96.5	96.4	96.5	96.5	96.5	96.4	91.3		
H7N7	Ref[e]	CY186001	92.0	92.0	91.9	92.0	92.0	91.9	92.0	91.9	91.9	91.9	91.9	91.9	99.6	99.6	99.6	99.6	99.5	99.6	91.8	96.8	
H9N1	Ref[f]	CY195824	97.1	97.1	97.0	97.1	97.1	97.0	97.0	97.0	97.0	97.0	97.0	97.0	91.7	91.7	91.7	91.7	91.7	91.7	96.7	91.7	91.9

[a] Early spring (March) recovered virues (ES); [b] Late spring (May) recovered viruses (LS); [c] Delaware Bay (May) recovered viruses (DB); [d] Virus AI12-1161 was collected in March 2012, subtype LP H5N9; [e] Reference sequence accession CY186001 strain name is A/ruddy turnstone/NJ/AI11-1678/2011/H7N7; [f] Reference sequence accession CY195824 strain name is A/gull/MA/13JR00943/2013/H9N1.

Supplemental Table S3. Pairwise distance matrix for 2,215 nucleotides of the PB1 gene (segment 2) for viruses analyzed in this study, and two reference sequences. Values are in percentages and shaded according to nucleotide identity: dark gray ≥ 99.0%.

			**Southeastern US viruses**												**Delaware Bay viruses**								**Ref**
**Subtype**	**Season**	**Virus ID**	**1161[d]**	**1244**	**1340**	**1368**	**1476**	**1541**	**1590**	**1591**	**1592**	**1593**	**1638**	**1683**	**2010**	**2120**	**2166**	**2210**	**2279**	**2356**	**2375**	**2871**	**CY186000**
H5N9	ES[a]	1244	100.0																				
H6N1	LS[b]	1340	99.5	99.5																			
H6N1	LS	1368	99.5	99.5	99.7																		
H5N9	ES	1476	100.0	100.0	99.5	99.5																	
H6N1	LS	1541	99.5	99.5	100.0	99.7	99.5																
H6N1	LS	1590	99.5	99.5	100.0	99.7	99.5	100.0															
H6N1	LS	1591	99.5	99.5	100.0	99.6	99.5	100.0	100.0														
H6N1	LS	1592	99.5	99.5	100.0	99.6	99.5	100.0	100.0	99.9													
H6N1	LS	1593	99.5	99.5	100.0	99.7	99.5	100.0	100.0	100.0	100.0												
H6N1	LS	1638	99.5	99.5	100.0	99.7	99.5	100.0	100.0	100.0	100.0	100.0											
H6N1	LS	1683	99.5	99.5	100.0	99.7	99.5	100.0	100.0	100.0	100.0	100.0	100.0										
H6N1	DB[c]	2010	99.6	99.6	99.3	99.3	99.6	99.3	99.3	99.2	99.2	99.3	99.3	99.3									
H6N1	DB	2120	99.7	99.7	99.4	99.4	99.7	99.4	99.4	99.3	99.3	99.4	99.4	99.4	99.7								
H6N1	DB	2166	96.0	96.0	95.7	95.9	96.0	95.7	95.7	95.7	95.7	95.7	95.7	95.7	95.8	95.8							
H6N1	DB	2210	96.0	96.0	95.7	95.9	96.0	95.7	95.7	95.7	95.7	95.7	95.7	95.7	95.8	95.8	100.0						
H5N9	DB	2279	99.7	99.7	99.4	99.4	99.7	99.4	99.4	99.4	99.4	99.4	99.4	99.4	99.5	99.6	95.8	95.8					
H6N1	DB	2356	99.6	99.6	99.3	99.3	99.6	99.3	99.3	99.3	99.3	99.3	99.3	99.3	100.0	99.8	95.8	95.8	99.5				
H6N1	DB	2375	93.3	93.3	93.1	93.1	93.3	93.1	93.1	93.1	93.1	93.1	93.1	93.1	93.1	93.1	93.1	93.1	93.2	93.2			
H5N3	DB	2871	93.8	93.8	93.7	93.8	93.8	93.7	93.7	93.6	93.6	93.7	93.7	93.7	93.6	93.7	93.9	93.9	93.7	93.6	96.4		
H7N7	Ref[e]	CY186000	99.6	99.6	99.3	99.3	99.6	99.3	99.3	99.2	99.2	99.3	99.3	99.3	99.4	99.5	96.2	96.2	99.5	99.4	93.5	94.1	
H9N1	Ref[f]	CY195823	95.8	95.8	95.5	95.7	95.8	95.5	95.5	95.5	95.5	95.5	95.5	95.5	95.6	95.6	99.4	99.4	95.7	95.7	92.8	93.6	92.8

[a] Early spring (March) recovered viruses (ES); [b] Late spring (May) recovered viruses (LS); [c] Delaware Bay (May) recovered viruses (DB); [d] Virus AI12-1161 was collected in March 2012, subtype LP H5N9; [e] Reference sequence accession CY186000 strain name is A/ruddy turnstone/NJ/AI11-1678/2011/H7N7; [f] Reference sequence accession CY195823 strain name is A/gull/MA/13JR00943/2013/H9N1.

Supplemental Table S4. Pairwise distance matrix for 2,183 nucleotides of the PA gene (segment 3) for viruses analyzed in this study, and two reference sequences. Values are in percentages and shaded according to nucleotide identity: dark gray ≥ 99.0%; medium gray ≥ 98.0%.

			**Southeastern US viruses**												**Delaware Bay viruses**								**Ref**
**Subtype**	**Season**	**Virus ID**	**1161[d]**	**1244**	**1340**	**1368**	**1476**	**1541**	**1590**	**1591**	**1592**	**1593**	**1638**	**1683**	**2010**	**2120**	**2166**	**2210**	**2279**	**2356**	**2375**	**2871**	**CY185999**
H5N9	ES[a]	1244	100.0																				
H6N1	LS[b]	1340	99.3	99.3																			
H6N1	LS	1368	99.4	99.4	99.9																		
H5N9	ES	1476	99.9	99.9	99.2	99.3																	
H6N1	LS	1541	99.3	99.3	100.0	99.9	99.2																
H6N1	LS	1590	99.3	99.3	100.0	99.9	99.2	100.0															
H6N1	LS	1591	99.3	99.3	100.0	99.9	99.2	100.0	100.0														
H6N1	LS	1592	99.3	99.3	100.0	99.9	99.2	100.0	100.0	100.0													
H6N1	LS	1593	99.3	99.3	100.0	99.9	99.2	100.0	100.0	100.0	100.0												
H6N1	LS	1638	99.3	99.3	100.0	99.9	99.2	100.0	100.0	100.0	100.0	100.0											
H6N1	LS	1683	99.3	99.3	100.0	99.9	99.2	100.0	100.0	100.0	100.0	100.0	100.0										
H6N1	DB[c]	2010	87.2	87.2	87.3	87.4	87.2	87.3	87.3	87.3	87.3	87.3	87.3	87.3									
H6N1	DB	2120	87.4	87.4	87.6	87.7	87.4	87.6	87.6	87.6	87.6	87.6	87.6	87.6	99.6								
H6N1	DB	2166	87.2	87.2	87.3	87.4	87.2	87.3	87.3	87.3	87.3	87.3	87.3	87.3	99.7	99.7							
H6N1	DB	2210	87.2	87.2	87.3	87.4	87.2	87.3	87.3	87.3	87.3	87.3	87.3	87.3	99.7	99.7	100.0						
H5N9	DB	2279	87.2	87.2	87.3	87.4	87.2	87.3	87.3	87.3	87.3	87.3	87.3	87.3	99.7	99.4	99.5	99.5					
H6N1	DB	2356	87.3	87.3	87.4	87.5	87.3	87.4	87.4	87.4	87.4	87.4	87.4	87.4	99.9	99.5	99.6	99.6	99.7				
H6N1	DB	2375	99.2	99.2	99.4	99.5	99.1	99.4	99.4	99.4	99.4	99.4	99.4	99.4	87.3	87.5	87.3	87.3	87.3	87.4			
H5N3	DB	2871	98.3	98.3	98.2	98.2	98.2	98.2	98.2	98.2	98.2	98.2	98.2	98.2	87.4	87.7	87.4	87.4	87.4	87.5	98.1		
H7N7	Ref[e]	CY185999	99.6	99.6	99.4	99.4	99.5	99.4	99.4	99.4	99.4	99.4	99.4	99.4	87.4	87.6	87.4	87.4	87.4	87.5	99.4	98.6	
H9N1	Ref[f]	CY195822	87.5	87.5	87.6	87.7	87.4	87.6	87.6	87.6	87.6	87.6	87.6	87.6	90.2	90.3	90.3	90.3	90.1	90.3	87.6	87.4	87.7

[a] Early spring (March) recovered viruses (ES); [b] Late spring (May) recovered viruses (LS); [c] Delaware Bay (May) recovered viruses (DB); [d] Virus AI12-1161 was collected in March 2012, subtype LP H5N9; [e] Reference sequence accession CY185999 strain name is A/ruddy turnstone/NJ/AI11-1678/2011/H7N7; [f] Reference sequence accession CY195822 strain name is A/gull/MA/13JR00943/2013/H9N1.

Supplemental Table S5. Pairwise distance matrix for 816 nucleotides of the NS gene (segment 8) for viruses analyzed in this study, and two reference sequences. Values are in percentages and shaded according to nucleotide identity: dark gray ≥ 99.0%; medium gray ≥ 98.0%; light gray ≥ 97.0%.

			Southeastern US viruses												Delaware Bay viruses								Ref
**Subtype**	**Season**	**Virus ID**	**1161[d]**	**1244**	**1340**	**1368**	**1476**	**1541**	**1590**	**1591**	**1592**	**1593**	**1638**	**1683**	**2010**	**2120**	**2166**	**2210**	**2279**	**2356**	**2375**	**2871**	**CY185995**
H5N9	ES[a]	1244	100.0																				
H6N1	LS[b]	1340	99.6	99.6																			
H6N1	LS	1368	99.5	99.5	99.9																		
H5N9	ES	1476	99.9	99.9	99.8	99.6																	
H6N1	LS	1541	99.6	99.6	100.0	99.9	99.8																
H6N1	LS	1590	99.6	99.6	100.0	99.9	99.8	100.0															
H6N1	LS	1591	99.6	99.6	100.0	99.9	99.8	100.0	100.0														
H6N1	LS	1592	99.6	99.6	100.0	99.9	99.8	100.0	100.0	100.0													
H6N1	LS	1593	99.6	99.6	100.0	99.9	99.8	100.0	100.0	100.0	100.0												
H6N1	LS	1638	99.6	99.6	100.0	99.9	99.8	100.0	100.0	100.0	100.0	100.0											
H6N1	LS	1683	99.6	99.6	100.0	99.9	99.8	100.0	100.0	100.0	100.0	100.0	100.0										
H6N1	DB[c]	2010	95.1	95.1	95.2	95.1	95.2	95.2	95.2	95.2	95.2	95.2	95.2	95.2									
H6N1	DB	2120	94.7	94.7	94.9	94.7	94.9	94.9	94.9	94.9	94.9	94.9	94.9	94.9	99.1								
H6N1	DB	2166	94.9	94.9	95.0	94.9	95.0	95.0	95.0	95.0	95.0	95.0	95.0	95.0	99.3	99.6							
H6N1	DB	2210	95.0	95.0	95.1	95.0	95.1	95.1	95.1	95.1	95.1	95.1	95.1	95.1	99.4	99.8	99.9						
H5N9	DB	2279	99.8	99.8	99.9	99.8	99.9	99.9	99.9	99.9	99.9	99.9	99.9	99.9	95.3	95.0	95.1	95.2					
H6N1	DB	2356	95.1	95.1	95.2	95.1	95.2	95.2	95.2	95.2	95.2	95.2	95.2	95.2	100.0	99.1	99.3	99.4	95.3				
H6N1	DB	2375	99.3	99.3	99.4	99.3	99.4	99.4	99.4	99.4	99.4	99.4	99.4	99.4	95.1	94.7	94.9	95.0	99.5	95.1			
H5N3	DB	2871	95.8	95.8	96.0	95.8	96.0	96.0	96.0	96.0	96.0	96.0	96.0	96.0	97.4	97.3	97.4	97.5	96.1	97.4	95.8		
H7N7	Ref[e]	CY185998	95.1	95.1	95.2	95.1	95.2	95.2	95.2	95.2	95.2	95.2	95.2	95.2	98.2	98.0	98.2	98.3	95.3	98.2	95.1	97.8	
H9N1	Ref[f]	CY195821	95.5	95.5	95.3	95.2	95.6	95.3	95.3	95.3	95.3	95.3	95.3	95.3	96.8	96.4	96.6	96.7	95.5	96.8	95.2	98.2	96.9

[a] Early spring (March) recovered virues (ES); [b] Late spring (May) recovered viruses (LS); [c] Delaware Bay (May) recovered viruses (DB); [d] Virus AI12-1161 was collected in March 2012, subtype LP H5N9; [e] Reference sequence accession CY185998 strain name is A/ruddy turnstone/NJ/AI11-1678/2011/H7N7; [f] Reference sequence accession CY195821 strain name is A/gull/MA/13JR00943/2013/H9N1.

Supplemental Table S6. Pairwise distance matrix for 1,438 nucleotides of the NP gene (segment 5) for viruses analyzed in this study, and two reference sequences. Values are in percentages and shaded according to nucleotide identity: dark gray ≥ 99.0%; medium gray ≥ 98.0%.

			Southeastern US viruses												Delaware Bay viruses								Ref
**Subtype**	**Season**	**Virus ID**	**1161[d]**	**1244**	**1340**	**1368**	**1476**	**1541**	**1590**	**1591**	**1592**	**1593**	**1638**	**1683**	**2010**	**2120**	**2166**	**2210**	**2279**	**2356**	**2375**	**2871**	**CY185997**
H5N9	ES[a]	1244	100.0																				
H6N1	LS[b]	1340	98.4	98.4																			
H6N1	LS	1368	92.1	92.1	91.9																		
H5N9	ES	1476	100.0	100.0	98.4	92.1																	
H6N1	LS	1541	98.4	98.4	100.0	91.9	98.4																
H6N1	LS	1590	98.3	98.3	99.9	91.9	98.3	99.9															
H6N1	LS	1591	98.4	98.4	100.0	91.9	98.4	100.0	99.9														
H6N1	LS	1592	98.4	98.4	100.0	91.9	98.4	100.0	99.9	100.0													
H6N1	LS	1593	98.4	98.4	100.0	91.9	98.4	100.0	99.9	100.0	100.0												
H6N1	LS	1638	98.4	98.4	100.0	91.9	98.4	100.0	99.9	100.0	100.0	100.0											
H6N1	LS	1683	98.4	98.4	100.0	91.9	98.4	100.0	99.9	100.0	100.0	100.0	100.0										
H6N1	DB[c]	2010	99.9	99.9	98.4	91.9	99.9	98.4	98.3	98.4	98.4	98.4	98.4	98.4									
H6N1	DB	2120	99.9	99.9	98.4	91.9	99.9	98.4	98.3	98.4	98.4	98.4	98.4	98.4	100.0								
H6N1	DB	2166	99.9	99.9	98.4	91.9	99.9	98.4	98.3	98.4	98.4	98.4	98.4	98.4	100.0	100.0							
H6N1	DB	2210	99.8	99.8	98.5	91.9	99.8	98.5	98.4	98.5	98.5	98.5	98.5	98.5	99.9	99.9	99.9						
H5N9	DB	2279	99.5	99.5	98.3	91.7	99.5	98.3	98.3	98.3	98.3	98.3	98.3	98.3	99.5	99.5	99.5	99.4					
H6N1	DB	2356	99.8	99.8	98.3	91.9	99.8	98.3	98.3	98.3	98.3	98.3	98.3	98.3	99.9	99.9	99.9	99.9	99.4				
H6N1	DB	2375	92.1	92.1	92.0	99.9	92.1	92.0	91.9	92.0	92.0	92.0	92.0	92.0	92.0	92.0	92.0	91.9	91.8	91.9			
H5N3	DB	2871	93.3	93.3	93.4	91.5	93.3	93.4	93.3	93.4	93.4	93.4	93.4	93.4	93.4	93.4	93.4	93.3	93.3	93.3	91.6		
H7N7	Ref[e]	CY185997	91.9	91.9	91.7	93.7	91.9	91.7	91.7	91.7	91.7	91.7	91.7	91.7	91.8	91.8	91.8	91.7	91.7	91.7	93.8	91.6	
H9N1	Ref[f]	CY195820	92.2	92.2	92.1	99.7	92.2	92.1	92.0	92.1	92.1	92.1	92.1	92.1	92.1	92.1	92.1	92.0	91.9	92.0	99.8	91.7	93.7

[a] Early spring (March) recovered viruses (ES); [b] Late spring (May) recovered viruses (LS); [c] Delaware Bay (May) recovered viruses (DB); [d] Virus AI12-1161 was collected in March 2012, subtype LP H5N9; [e] Reference sequence accession CY185997 strain name is A/ruddy turnstone/NJ/AI11-1678/2011/H7N7; [f] Reference sequence accession CY195820 strain name is A/gull/MA/13JR00943/2013/H9N1.

Supplemental Table S7. Pairwise distance matrix for 928 nucleotides of the matrix gene (segment 7) for viruses analyzed in this study, and two reference sequences. Values are in percentages and shaded according to nucleotide identity: dark gray ≥ 99.0%; medium gray ≥ 98.0%; light gray ≥ 97.0%.

			Southeastern US viruses												Delaware Bay viruses								Ref
**Subtype**	**Season**	**Virus ID**	**1161[d]**	**1244**	**1340**	**1368**	**1476**	**1541**	**1590**	**1591**	**1592**	**1593**	**1638**	**1683**	**2010**	**2120**	**2166**	**2210**	**2279**	**2356**	**2375**	**2871**	**CY185995**
H5N9	ES[a]	1244	100.0																				
H6N1	LS[b]	1340	96.0	96.0																			
H6N1	LS	1368	96.0	96.0	100.0																		
H5N9	ES	1476	100.0	100.0	96.0	96.0																	
H6N1	LS	1541	96.0	96.0	100.0	100.0	96.0																
H6N1	LS	1590	96.0	96.0	100.0	100.0	96.0	100.0															
H6N1	LS	1591	96.0	96.0	100.0	100.0	96.0	100.0	100.0														
H6N1	LS	1592	96.0	96.0	100.0	100.0	96.0	100.0	100.0	100.0													
H6N1	LS	1593	96.0	96.0	100.0	100.0	96.0	100.0	100.0	100.0	100.0												
H6N1	LS	1638	96.0	96.0	100.0	100.0	96.0	100.0	100.0	100.0	100.0	100.0											
H6N1	LS	1683	96.0	96.0	100.0	100.0	96.0	100.0	100.0	100.0	100.0	100.0	100.0										
H6N1	DB[c]	2010	96.4	96.4	99.4	99.4	96.4	99.4	99.4	99.4	99.4	99.4	99.4	99.4									
H6N1	DB	2120	96.6	96.6	99.5	99.5	96.6	99.5	99.5	99.5	99.5	99.5	99.5	99.5	99.9								
H6N1	DB	2166	100.0	100.0	96.0	96.0	100.0	96.0	96.0	96.0	96.0	96.0	96.0	96.0	96.4	96.6							
H6N1	DB	2210	100.0	100.0	96.0	96.0	100.0	96.0	96.0	96.0	96.0	96.0	96.0	96.0	96.4	96.6	100.0						
H5N9	DB	2279	96.6	96.6	99.2	99.2	96.6	99.2	99.2	99.2	99.2	99.2	99.2	99.2	99.7	99.8	96.6	96.6					
H6N1	DB	2356	96.6	96.6	99.5	99.5	96.6	99.5	99.5	99.5	99.5	99.5	99.5	99.5	99.9	100.0	96.6	96.6	99.8				
H6N1	DB	2375	99.9	99.9	95.9	95.9	99.9	95.9	95.9	95.9	95.9	95.9	95.9	95.9	96.3	96.4	99.9	99.9	96.4	96.4			
H5N3	DB	2871	97.4	97.4	96.2	96.2	97.4	96.2	96.2	96.2	96.2	96.2	96.2	96.2	96.4	96.6	97.4	97.4	96.6	96.6	97.3		
H7N7	Ref[e]	CY185995	96.9	96.9	99.1	99.1	96.9	99.1	99.1	99.1	99.1	99.1	99.1	99.1	99.6	99.7	96.9	96.9	99.5	99.7	96.8	96.9	
H9N1	Ref[f]	CY195818	96.4	96.4	96.7	96.7	96.4	96.7	96.7	96.7	96.7	96.7	96.7	96.7	97.1	97.2	96.4	96.4	97.0	97.2	96.3	96.6	97.5

[a] Early spring (March) recovered virues (ES); [b] Late spring (May) recovered viruses (LS); [c] Delaware Bay (May) recovered viruses (DB); [d] Virus AI12-1161 was collected in March 2012, subtype LP H5N9; [e] Reference sequence accession CY185995 strain name is A/ruddy turnstone/NJ/AI11-1678/2011/H7N7; [f] Reference sequence accession CY195818 strain name is A/gull/MA/13JR00943/2013/H9N1.

Supplemental Table S8. Pairwise distance matrix for (a) 1,622 nucleotides of the HA6 gene (segment 4) and (b) 1,691 nucleotides of the HA5 gene for viruses analyzed in this study. Values are in percentages and shaded according to nucleotide identity: dark gray ≥ 99.0%; medium gray ≥ 98.0%; light gray ≥ 97.0%.

**7A**			**Southeastern US viruses**									**Delaware Bay viruses**				
**Subtype**	**Season**	**Virus ID**	**1340[c]**	**1368**	**1541**	**1590**	**1591**	**1592**	**1593**	**1638**	**1683**	**2010**	**2120**	**2166**	**2210**	**2356**
H6N1	LS[a]	1368	99.9													
H6N1	LS	1541	100.0	99.9												
H6N1	LS	1590	100.0	99.9	100.0											
H6N1	LS	1591	99.9	99.8	99.9	99.9										
H6N1	LS	1592	99.9	99.8	99.9	99.9	100.0									
H6N1	LS	1593	100.0	99.9	100.0	100.0	99.9	99.9								
H6N1	LS	1638	99.9	99.8	99.9	99.9	99.9	99.9	99.9							
H6N1	LS	1683	99.9	99.8	99.9	99.9	99.9	99.9	99.9	99.9						
H6N1	DB[b]	2010	99.6	99.4	99.6	99.6	99.5	99.5	99.6	99.5	99.5					
H6N1	DB	2120	99.6	99.4	99.6	99.6	99.5	99.5	99.6	99.6	99.5	99.8				
H6N1	DB	2166	99.5	99.4	99.5	99.5	99.4	99.4	99.5	99.4	99.4	99.7	99.7			
H6N1	DB	2210	99.5	99.4	99.5	99.5	99.4	99.4	99.5	99.4	99.4	99.7	99.7	100.0		
H6N1	DB	2356	99.6	99.4	99.6	99.6	99.5	99.5	99.6	99.5	99.5	99.9	99.8	99.7	99.7	
H6N1	DB	2375	99.2	99.1	99.2	99.2	99.1	99.1	99.1	99.1	99.1	99.4	99.4	99.3	99.3	99.4

[a] Late spring (May) recovered viruses (LS); [b] Delaware Bay (May) recovered viruses (DB); [c] Virus AI12-1340 was collected in May 2012, subtype H6N1.

**7B**			**Southeastern US viruses**			**Delaware Bay virus**
**Subtype**	**Season**	**Virus ID**	**1161[c]**	**1244**	**1476**	**2279**
H5N9	ES[a]	1244	99.9			
H5N9	ES	1476	99.8	99.9		
H5N9	DB[b]	2279	99.4	99.4	99.3	
H5N3	DB	2871	97.7	97.7	97.7	97.5

[a] Early spring (March) recovered viruses (ES); [b] Delaware Bay (May) recovered viruses (DB); [c] Virus AI12-1161 was collected in March 2012, subtype LP H5N9

Supplemental Table S9. Pairwise distance matrix for (a) 1,395 nucleotides of the NA1 gene (segment 6) and (b) 1,409 nucleotides of the NA9 gene for viruses analyzed in this study and one reference sequence. Values are in percentages and shaded according to nucleotide identity: dark gray, ≥ 99.0%.

**8A**			**overwintering viruses**									**Delaware Bay viruses**					
**Subtype**	**Season**	**Virus ID**	**1340[c]**	**1368**	**1541**	**1590**	**1591**	**1592**	**1593**	**1638**	**1683**	**2010**	**2120**	**2166**	**2210**	**2356**	**2375**
H6N1	LS[a]	1368	99.9														
H6N1	LS	1541	100.0	99.9													
H6N1	LS	1590	100.0	99.9	100.0												
H6N1	LS	1591	100.0	99.9	100.0	100.0											
H6N1	LS	1592	100.0	99.9	100.0	100.0	100.0										
H6N1	LS	1593	100.0	99.9	100.0	100.0	99.9	99.9									
H6N1	LS	1638	99.9	99.9	99.9	99.9	99.9	99.9	99.9								
H6N1	LS	1683	100.0	99.9	100.0	100.0	100.0	100.0	100.0	99.9							
H6N1	DB[b]	2010	99.6	99.6	99.6	99.6	99.6	99.6	99.6	99.6	99.6						
H6N1	DB	2120	99.6	99.5	99.6	99.6	99.6	99.6	99.6	99.5	99.6	99.9					
H6N1	DB	2166	99.5	99.4	99.5	99.5	99.5	99.5	99.5	99.4	99.5	99.9	99.8				
H6N1	DB	2210	99.6	99.5	99.6	99.6	99.6	99.6	99.6	99.5	99.6	99.9	99.9	99.9			
H6N1	DB	2356	99.6	99.6	99.6	99.6	99.6	99.6	99.6	99.6	99.6	100.0	99.9	99.9	99.9		
H6N1	DB	2375	93.0	92.9	93.0	93.0	93.0	93.0	93.0	92.9	93.0	92.8	92.7	92.6	92.7	92.8	
H9N1	Ref[d]	CY195824	92.8	92.7	92.8	92.8	92.8	92.8	92.8	92.7	92.8	92.5	92.5	92.4	92.5	92.5	99.4

[a] Late spring (May) recovered viruses (LS); [b] Delaware Bay (May) recovered viruses (DB); [c] Virus AI12-1340 was collected in late spring (LS), subtype H6N1;
[d] Reference sequence accession CY195824 strain name is A/gull/MA/13JR00943/2013/H9N1.

**8B**			**overwintering viruses**		
**Subtype**	**Season**	**Virus ID**	**1161[c]**	**1244**	**1476**
H5N9	ES[a]	1244	100.0		
H5N9	ES	1476	100.0	100.0	
H5N9	DB[b]	2279	99.3	99.3	99.3

[a] Early spring (March) recovered viruses (ES); [b] Delaware Bay recovered virus (DB); [c] Virus AI12-1161 is an early spring (ES) virus, subtype LP H5N9.
